# Powassan Virus Infection Detected by Metagenomic Next-Generation Sequencing, Ohio, USA

**DOI:** 10.3201/eid2904.221005

**Published:** 2023-04

**Authors:** Marjorie Farrington, Jamie Elenz, Matthew Ginsberg, Charles Y. Chiu, Steve Miller, Scott F. Pangonis

**Affiliations:** Children’s Hospital Medical Center of Akron, Akron, Ohio, USA (M. Farrington, M. Ginsberg, S.F Pangonis);; Columbiana County Health District, Lisbon, Ohio, USA (J. Elenz);; University of California, San Francisco, California, USA (C.Y. Chiu, S. Miller)

**Keywords:** Powassan virus, vector-borne infections, viruses, zoonoses, tickborne infection, emerging infections, encephalitis, Ohio, United States

## Abstract

We describe a 4-year-old male patient in Ohio, USA, who had encephalitis caused by Powassan virus lineage 2. Virus was detected by using metagenomic next-generation sequencing and confirmed with IgM and plaque reduction neutralization assays. Clinicians should recognize changing epidemiology of tickborne viruses to enhance encephalitis diagnosis and management.

Powassan virus (POWV) is a tickborne flavivirus that causes encephalitis with severe neurologic sequelae (*1*). In the United States, POWV infections occur primarily in the Northeast and Great Lakes regions (*2*). We report a case of human POWV infection in Ohio.

A 4-year-old boy was brought to the emergency department because of fever, vomiting, and convulsive status epilepticus. He had no neurologic history or developmental delays. Mosquito and tick exposure history was substantial, although no engorged ticks were recently removed. The patient had not traveled outside of Ohio. 

Results of a computed tomography scan of the head were unremarkable. We initiated intravenous vancomycin, ceftriaxone, and acyclovir. Magnetic resonance imaging showed left temporal pulvinar and thalamic T2-weighted fluid attenuated inversion recovery hyperintensity and restricted diffusion; an electroencephalogram showed lateralized periodic discharges. Cerebrospinal fluid (CSF) was collected by lumbar puncture, revealing a leukocyte count of 44 cells/μL (reference range <10 cells/μL) of which 85% were lymphocytes; glucose and protein levels were normal. The patient’s BIOFIRE FILMARRAY Meningitis/Encephalitis PCR panel (bioMérieux, https://www.biomerieux-diagnostics.com) was negative (Table). He was admitted to the pediatric intensive care unit, and seizures were controlled with anticonvulsants. Tests for infectious and noninfectious causes of meningitis and encephalitis were negative (Table). Antimicrobial drugs were discontinued after negative bacterial cultures were observed. Acyclovir was discontinued after PCR of CSF for herpes simplex virus was negative. 

**Table:** Test results for infectious and noninfectious causes of meningitis and encephalitis in study of Powassan virus infection detected by metagenomic next-generation sequencing, Ohio, USA*

Tests	Results
Infectious causes	
CSF meningitis encephalitis PCR panel†	No organisms detected

CSF bacterial culture	Negative
Lyme disease serology	Negative
Serum arbovirus IgG and IgM panel, reference range <1:10‡	<1:10
EBV viral capsid antigen IgM and IgG, antibody index <0.2‡	Negative
EBV nuclear antigen IgG, antibody index <0.2‡	Negative

*Bartonella henselae* and *B*. *quintana* antibodies	
IgG, <1:128	Negative
IgM, <1:20	Negative

Noninfectious causes	
Serum antinuclear antibody	Negative
Serum myelin oligodendrocyte glycoprotein	Negative

*CSF, cerebrospinal fluid; EBV, Epstein-Barr virus. †BIOFIRE FILMARRAY Meningitis/Encephalitis PCR panel (bioMérieux, https://www.biomerieux-diagnostics.com). Panel included *Streptococcus pneumoniae, S*. *agalactiae, Haemophilus influenzae, Neisseria meningitidis, Listeria monocytogenes, Escherichia coli*, *Cryptococcus neoformans/gattii*, varicella-zoster virus*,* cytomegalovirus*,* herpes simplex virus types 1 and 2*, Enterovirus,* human herpesvirus 6*,* and *Parechovirus*. ‡Acute and convalescent serum samples were sent to Mayo Clinic Laboratories (https://www.mayocliniclabs.com) for arbovirus and EBV analysis. Arbovirus panel included La Crosse encephalitis virus, eastern equine encephalitis virus, western equine encephalitis virus, West Nile encephalitis virus, and St. Louis encephalitis virus.

On hospitalization day 5, severe neurologic decline developed, and brain magnetic resonance imaging was repeated. New areas of T2 hyperintensity and restricted diffusion and thalamic microhemorrhages in a rhombencephalitis pattern were identified. Lumbar puncture was repeated, revealing considerable lymphocytic pleocytosis and elevated protein (156 mg/dL). Leading diagnoses were autoimmune encephalitis and acute necrotizing encephalopathy of childhood (ANEC). The patient exhibited severe encephalopathy, nystagmus, right hemiparesis, and diffuse hypertonia. He was treated with high dose methylprednisolone, plasmapheresis, and intravenous immunoglobulins. 

Genetic testing for familial ANEC type 1 was negative. We sent CSF obtained on hospital day 5 to the University of California San Francisco for metagenomic next-generation sequencing (mNGS), which detected a single 140-nt POWV sequence (Appendix). We performed phylogenetic analysis of the sequence and assigned the patient’s virus to lineage 2 (Figure, panel A); the sequence mapped to the nonstructural *NS3* gene (Figure, panel B). We performed BLAST (*3*) sequence alignments in GenBank, which yielded matches to POWV genomes (Figure, panel C). CSF samples obtained before the patient received intravenous Igs were sent for confirmatory testing to the CDC Arbovirus Diagnostic Laboratory, Division of Vector-Borne Diseases, National Center for Emerging and Zoonotic Infectious Diseases (https://www.cdc.gov/ncezid/dvbd/specimensub), which showed IgM against POWV (capture ELISA), negative POWV-specific PCR, and a positive plaque reduction neutralization test at 1:8 dilution (reference range <1:2), confirming the final diagnosis was ANEC caused by POWV. The patient continued having severe neurologic sequalae requiring a tracheostomy, gastrostomy tube, and inpatient rehabilitation. At follow-up 1 year after admission, he had been decannulated, was able to orally ingest liquids and solids, and ambulated independently, but he had substantial language and cognitive deficits. 

**Figure:**
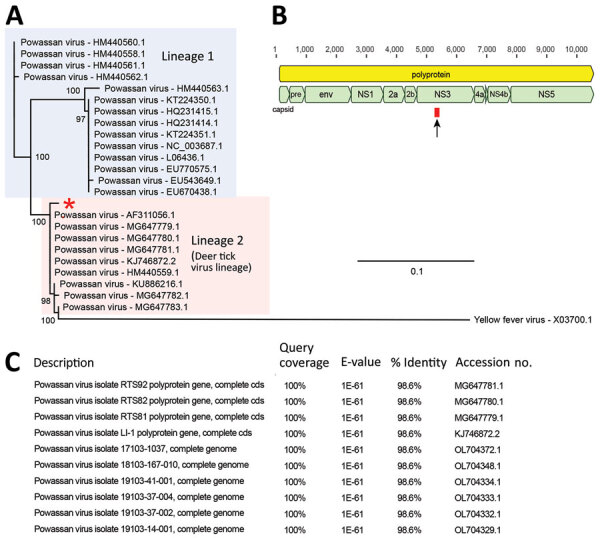
Identification and phylogenetic analysis of Powassan virus found in cerebrospinal fluid of 4-year-old boy detected by metagenomic next-generation sequencing, Ohio, USA. A) Phylogenetic analysis of the 140-bp region in the Powassan virus genome corresponding to the single sequence read detected by metagenomic next-generation sequencing. Single read from the patient in this study was aligned with sequences from 23 representative Powassan virus genomes from lineage 1 (blue shaded box) and lineage 2 (deer tick virus lineage, pink shaded box) and 1 yellow fever virus sequence as an outgroup by using MAFFT v7.388 (Appendix reference *11* ). Phylogenetic tree was constructed by using the maximum-likelihood method and PhyML 3.0 software (Appendix reference *12*); support values for the main branches are shown. Powassan virus from our patient (red asterisk) belongs to lineage 2. GenBank accession numbers are shown for each sequence. Scale bar indicates nucleotide substitutions per site. B) Powassan virus genome showing major capsid and nonstructural genes. Single sequence read from the patient mapped to the *NS3* gene (arrow and red box). C) List of top 10 GenBank reference sequences matching the patient’s 140-nt read after using MegaBLAST (https://blast.ncbi.nlm.nih.gov) alignment as default setting, each showing 98.6% sequence identity. If Powassan virus sequences were excluded from the reference database, no other matches in GenBank were found. Cds, coding sequence; env, envelope protein; NS, nonstructural; pre, M protein precursor peptide.

Diagnostic yield for patients with encephalitis from any cause is 37% (*4*); mNGS enables detection of nearly all pathogens in a single assay and can improve diagnostic yield for patients with CNS infections (*5*). We identified 1 nucleotide sequence aligning to POWV, which is below the preestablished reporting threshold for a positive result of >3 reads spanning >3 regions of the viral genome (*5*). Thus, our finding was potentially a false positive, especially because POWV is not endemic to Ohio. However, POWV has never been a contaminant in clinical mNGS analyses of >4,000 CSF samples at the University of California San Francisco laboratory, and our result was confirmed as a true positive by a plaque reduction neutralization test**.** Of note, a case of POWV encephalitis identified by mNGS from 10 genomic sequencing reads has been reported (*6*). Furthermore, POWV-specific PCR of our patient’s CSF was negative, consistent with a low viral titer near the limit of detection by molecular assays. Patients with POWV encephalitis are often PCR negative because viremia precedes CNS disease development (*7*).

The number of POWV cases reported has increased over the past few decades (*2*), likely because of tick range expansion. *Ixodes scapularis* is the only tick species in northeastern Ohio that can transmit POWV (*8*). Tick range expansion might be caused by increasing temperatures from climate change (*9*), migration of animal hosts, and changes in land use or host populations (*10*). Ticks carrying POWV lineage 2 were not identified on the family’s property. However, healthcare and public health workers should be aware of changing epidemiology and potential emerging tickborne infections in nonendemic regions.

In conclusion, we identified a POWV infection in Ohio by using mNGS. Tests for autoimmune etiologies, familial ANEC (type 1), and other viral agents were negative, excluding alternative diagnoses. Our case highlights the ability of mNGS to identify rare or unexpected pathogens that cause encephalitis. Providers should recognize changing epidemiology of tickborne viruses, such as POWV, to enhance encephalitis diagnosis and management. When cases are identified, local public health departments should complete comprehensive entomological and epidemiologic studies to determine virus prevalence.

Appendix Additional information for Powassan virus infection detected by metagenomic next-generation sequencing, Ohio, USA.
