# Supplementary material for: Powassan Virus Infection Detected by Metagenomic Next-Generation Sequencing, Ohio, USA
Source: Emerg Infect Dis. 2023 Apr;29(4):838–41. doi: 10.3201/eid2904.221005 (PMC10045675; doi:10.3201/eid2904.221005)
Supplement: Appendix — Additional information for Powassan virus infection detected by metagenomic next-generation sequencing, Ohio, USA. [file 22-1005-Techapp-s1.pdf]

Article DOI: https://doi.org/10.3201/eid2904.221005
EID cannot ensure accessibility for supplementary materials supplied by authors. Readers who have difficulty accessing supplementary content should contact the authors for assistance.

Powassan Virus Infection Detected by Metagenomic Next-Generation Sequencing, Ohio, USA

Appendix

A single 140-nt sequence from Powassan virus was detected by metagenomic next-generation sequencing in the cerebrospinal fluid of a 4-year-old boy.

>NDX550313_RUO:156:HKNFTBGXK:1:22109:2530:9011#ATCTCAGG+ATAGCCTT/1

CAGCATCACAGTCCTGGACATGCATCCTGGAGCGGGAAAGACACACAGAGTCCTTC
CCGAGCTGATTCGTGAATGCATTGACAAAAGATTGAGGACTGTTGTGTTGGCCCCAA
CGCGGGTTGTTCTGAAAGAGATGGAAA

Appendix References

11. Katoh K, Standley DM. MAFFT: iterative refinement and additional methods. Methods Mol Biol. 2014;1079:131–46. PubMed https://doi.org/10.1007/978-1-62703-646-7_8

12. Guindon S, Dufayard J-F, Lefort V, Anisimova M, Hordijk W, Gascuel O. New algorithms and methods to estimate maximum-likelihood phylogenies: assessing the performance of PhyML 3.0. Syst Biol. 2010;59:307–21. PubMed https://doi.org/10.1093/sysbio/syq010
